# DBnorm as an R package for the comparison and selection of appropriate statistical methods for batch effect correction in metabolomic studies

**DOI:** 10.1038/s41598-021-84824-3

**Published:** 2021-03-11

**Authors:** Nasim Bararpour, Federica Gilardi, Cristian Carmeli, Jonathan Sidibe, Julijana Ivanisevic, Tiziana Caputo, Marc Augsburger, Silke Grabherr, Béatrice Desvergne, Nicolas Guex, Murielle Bochud, Aurelien Thomas

**Affiliations:** 1 Unit of Forensic Toxicology and Chemistry, CURML, Lausanne University Hospital-Geneva University Hospitals, Lausanne-Geneva, Switzerland; 2 Faculty Unit of Toxicology, CURML, Lausanne University Hospital, Faculty of Biology and Medicine, University of Lausanne, Lausanne, Switzerland; 3 Center for Primary Care and Public Health (Unisanté), University of Lausanne, Lausanne, Switzerland; 4 Population Health Laboratory, Department of Medicine and Public Health, University of Fribourg, Fribourg, Switzerland; 5 Unit of Metabolomics, Faculty of Biology and Medicine, University of Lausanne, Lausanne, Switzerland; 6 Center for Integrative Genomics, University of Lausanne, Lausanne, Switzerland; 7 CURML, Lausanne University Hospital-Geneva University Hospitals, Lausanne-Geneva, Switzerland; 8 BioInformatics Competence Center, University of Lausanne, Lausanne, Switzerland

**Keywords:** Metabolomics, Data processing, Statistical methods

## Abstract

As a powerful phenotyping technology, metabolomics provides new opportunities in biomarker discovery through metabolome-wide association studies (MWAS) and the identification of metabolites having a regulatory effect in various biological processes. While mass spectrometry-based (MS) metabolomics assays are endowed with high throughput and sensitivity, MWAS are doomed to long-term data acquisition generating an overtime-analytical signal drift that can hinder the uncovering of real biologically relevant changes. We developed “*dbnorm*”, a package in the R environment, which allows for an easy comparison of the model performance of advanced statistical tools commonly used in metabolomics to remove batch effects from large metabolomics datasets. “*dbnorm*” integrates advanced statistical tools to inspect the dataset structure not only at the macroscopic (sample batches) scale, but also at the microscopic (metabolic features) level. To compare the model performance on data correction, “*dbnorm*” assigns a score that help users identify the best fitting model for each dataset. In this study, we applied “*dbnorm*” to two large-scale metabolomics datasets as a proof of concept. We demonstrate that “*dbnorm*” allows for the accurate selection of the most appropriate statistical tool to efficiently remove the overtime signal drift and to focus on the relevant biological components of complex datasets.

## Introduction

Large datasets generated by metabolomics have become an essential source of information about molecular phenotypes in systems biology studies^1–4^. As an intermediate molecular layer between genes and disease phenotypes, metabolite levels and the corresponding pattern of changes are strongly associated with the degree of perturbation of biological systems and rewired metabolic networks for a given phenotype^5–12^.

Liquid chromatography-MS (LC–MS) is widely used in the study of the metabolome owing to the progressive improvement of its instrumental conditions in terms of its sensitivity and selectivity^13^. However, LC–MS-based metabolomics assays suffer from inherent variations in the distribution of signal measurements and/or in its signal sensitivity and intensity driven by external factors^14^. This signal drift is a major limitation of current data normalization in biomedical and clinical studies, resulting in unavoidable technical variations introduced during sample preparation and analysis^15^. In particular, such a drift can significantly compromise the technical precision and signal stability in large-scale studies, where the data acquisition for several hundreds to thousands of biological samples needs to be completed in different analytical blocks (i.e., batches) over several weeks or even months^15,16^. In this case, the largest variance in the dataset may be assigned to the batch effects or experimental run order, thus hindering identification of the real biological differences and true functional signals, leading to data misinterpretation^15,17,18^. Therefore, large metabolomics datasets need to be corrected for unwanted and within and between-batch, analytical variation to make the data comparable and to reveal biologically relevant changes^19–21^.

Several methods have been developed for the batch correction of metabolomics datasets, but the choice is not easy^22^. In most LC–MS-based metabolomics data processing workflows, signal intensity drift correction is performed using quality control (QC) samples. QC samples are aliquots of a QC pool, representative of the entire sample set. QC samples are injected within each batch to monitor the signal drift over time and to evaluate the system performance and data quality^23–25^. However, in large-scale studies, the preparation of such QC samples may be difficult due to the large number of samples, whose handling would involve additional freeze–thaw cycles. According to the experimental design surrogate QC samples can be applied^23,26–29^. In general, drift correction using QCs is based on the assumption that the same sources of variation apply to the metabolites present in both the biological samples and the QCs as their representative pools. In a majority of QC sample-based workflows, several commonly proposed algorithms are used for batch effect correction, such as feature-based correction algorithms^30^, regression-based models^30,31^, linear and nonlinear smoothing algorithms (e.g., *lowess* models)^23,31–34^, and classification- and regression-based models (i.e., random forest)^35^.

Models not based on QC samples mainly fall into two categories, matrix factorization (MF) and location-scale (L/S)-based methods, leveraging genomics-based approaches. Both of these methods can directly remove batch effects from the subject samples^36^. Initially, MF-based methods were developed to model the covariates in a differential analysis of the genomics data^37^. To remove batch variance, MF approaches usually perform either singular value decomposition (SVD) or principal component analysis (PCA), and the batch effect must be associated with the first factor (or component) showing the highest possible variance^38^. Some implementations of such an approach are independent component analysis (ICA) and EigenMS^36^ . The major drawback of the MF-based model is that if no clear batch effect is captured in the first ranked components, then the data matrix remains unchanged ^37^. Location-scale (L/S)-based methods are the most straightforward. Various implementations of such categories have been proposed, such as the mean-centering, standardization and ratio-based methods^38^ .

*ComBat* is also a function established in this category that uses the empirical Bayes methods (EB) framework for estimation of the L/S parameters that represent batch effects^18,39–42^. *ComBat* has the flexibility to adjust the batch effect by integrating information on both batches and the biological covariates or just in the batches if the demography of the population is not available^10,15^. This statistical approach is developed in the context of “parametric shrinkage adjustment”, in which the batch effect is removed in three steps: data standardization, batch effect estimation via empirical priors and batch effect removal using the adjusted estimators^42^. Although it makes strong parametric estimation, *ComBat* has been recognized for its superior performance in adjusting of unwanted variation compared to several other models^39^. Another statistical model that was originally developed for microarray data is *ber*, which removes batch effects by using linear fitting for both location and scale (L/S) parameters^43^. Interestingly, this model showed a better performance in microarray data compared to the empirical Bayes models^43^, but it was never tested on metabolomics data.

Although all of these approaches may be successfully applied to remove batch effects from metabolomics datasets, the selection of the most appropriate approach is dependent on the dataset structure and should be tested by the users^44^. With this goal, here we describe “*dbnorm*”, a new package in the R environment, which aims to compare state-of-the-art statistical approaches for the removal of batch effects from metabolomics datasets. This package incorporates several tools for preprocessing large datasets and estimation of missing values. In addition, “*dbnorm*” includes two distinct statistical models for batch effect correction: *ComBat*, both the *parametric* and *nonparametric* versions), and *ber,* including its *bagging* version, as explained in detail in the Methods section. Of note, for each analyzed dataset, “*dbnorm*” generates diagnostic plots that help users visualize the structure of their data at both the sample level and the feature level. Moreover, “*dbnorm*” calculates a score value that allows users to easily choose the statistical correction model that best fits their data structure. This score, which is denoted by adjusted- R squared (adj-R^2^), ranges from 0 to 1 and notifies quickly the highest linear association between metabolite levels and batch of analysis, as calculated based on adjusted coefficient of determination. The implementation of the “*dbnorm*” package is publicly available at https://github.com/NBDZ/dbnorm. In this report, we applied “*dbnorm*” to two large-scale metabolomics datasets as a proof of concept. We showed the properties of “*dbnorm*” to help users select the most efficient statistical tool to remove batch effects in metabolomics datasets and to thereby detect the relevant biological components of complex metabolomics datasets.

## Results

### Example analysis 1: batch effect correction in a large-scale targeted metabolomics dataset with *“dbnorm”*

The first dataset on which “*dbnorm*” was tested is a targeted measurement of the plasma metabolome of 1,079 individuals in the human SKIPOGH cohort. These samples were analyzed in 11 analytical batches over a period of 12 months, together with 135 QC samples that were injected every 10 samples (see Methods section). Data were generated in both positive and negative ionization modes, and 239 metabolites have been detected across all samples.

### Assessment and correction of the batch effect with “***dbnorm***”

**Figure 1:**
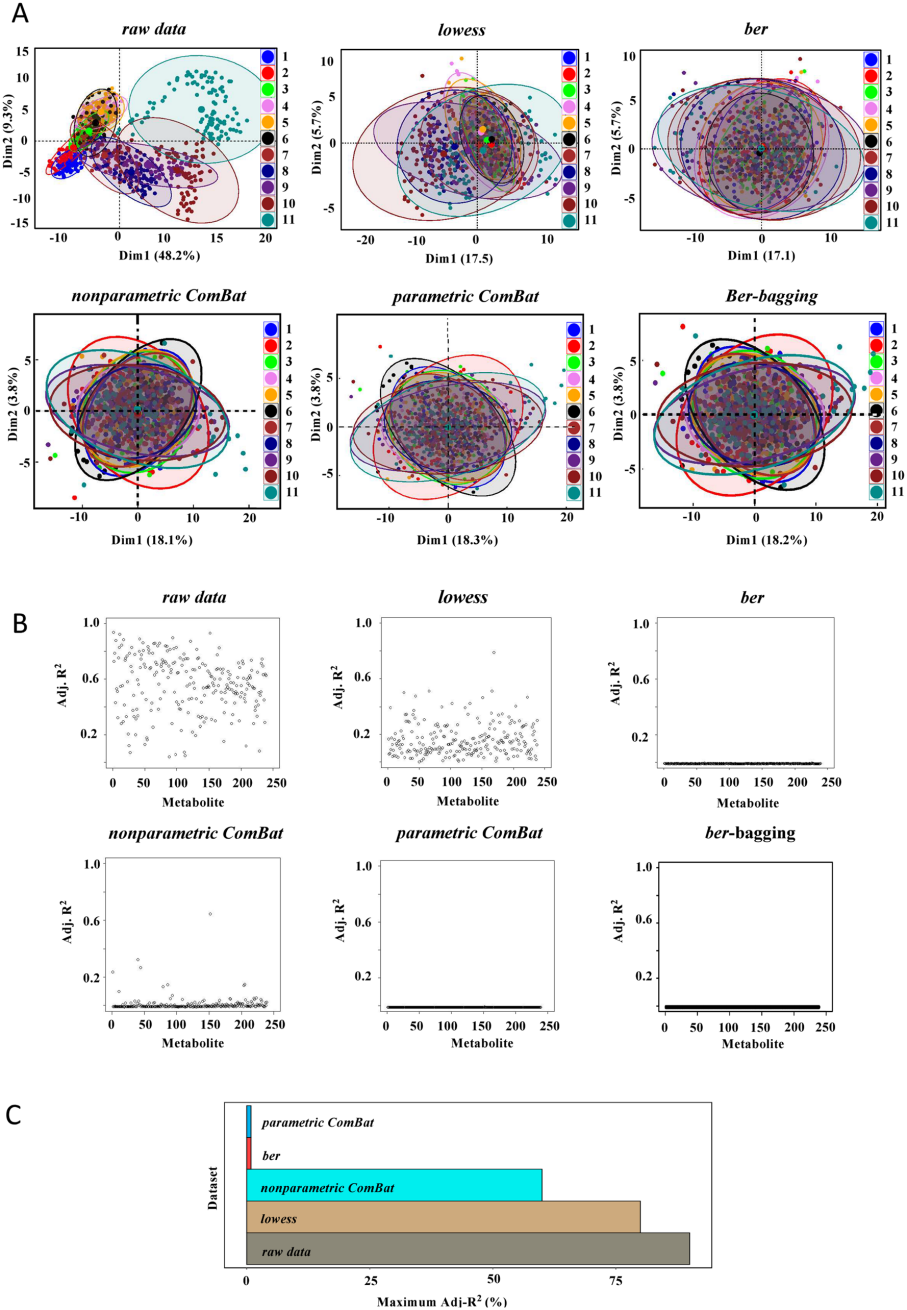
Monitoring of batch effect in the LC–MS targeted metabolomics analysis of the SKIPOGH human cross-sectional study with *“dbnorm”*. 1079 plasma samples were analyzed in 11 analytical batches over a period of 12 months. 239 metabolites were detected. (**A**) PCA of *raw* data shows separation of sample clusters mainly triggered by batch order. PCAs of *lowess*-, *ber*-, *nonparametric ComBat-*, *parametric ComBat-*, and *bagging ber*-corrected data showed decreased distinction between samples analyzed in the different batches. (**B**) Adjusted Coefficient of Determination (adj-R^2^), as estimated by regression model. In *raw* data metabolite intensities were strongly associated to batch level, some residual association was observed in *lowess*- and *nonparametric ComBat*-corrected data, while no association was present in *parametric ComBat*-, *ber*- and *bagging ber*-corrected data. (**C**) Score performance calculation for the correction of the signal drift across batches: the maximum absolute adj-R^2^, as estimated by regression model, is calculated for each dataset and presented as a bar plot. The lower the score, the higher the performance of the statistical model.

“*dbnorm*” was first employed to perform an explorative statistical analysis of the results obtained in this dataset. The Principal Component Analysis (PCA) plot of *raw* data generated by “*dbnorm*” showed that the samples were clustered by batch order (Fig. 1A). In parallel, the adjusted coefficient of determination (adj-R squared (R^2^)) revealed that the variability in the dataset was highly dependent on the across-batch signal drift, with > 50% of the variability explained by batch for most metabolites (Fig. 1B and Supplementary Table 1).

We thus employed the different statistical models implemented in the “*dbnorm*” R package to remove the batch effect observed in the entire dataset. To assess the efficiency of the “*dbnorm*” statistical methods, we also compared their performance with that of a reference QC sample-based model, such as *lowess*. PCA plots showed the spatial separation predictive of the batch effect, which was significantly reduced after correction of the data with all of the tested models (Fig. 1A). By applying all types of correction algorithms, the variability associated with each detected feature was strongly reduced, particularly with the two *ber* models and *parametric ComBat* (Fig. 1B), while with *nonparametric ComBat*, several metabolites still showed some residual variability. Of note, for each statistical model employed, “*dbnorm*” calculates a score, whose range is between 0 and 1, which is based on the highest adjusted-R squared (adj-R^2^). This simple measure of what percentage of variance (scaled to 100) of a dependent variable (i.e., metabolic feature) is explained by an independent variable (i.e., batch) prior to and after applying “*dbnorm*”, allows the user to quickly identify the statistical model that better fits the dataset. In addition, based on the proportion of variability explained by batch, “*dbnorm*” suggests the best compromise for correction of batch effects, also considering the consistency of overall model performance for all detected metabolites (see the Methods section). As shown in Fig. 1C, in our cohort study, the maximum variability detected for a metabolite was 0.78 (78%) for the *lowess*-corrected dataset (Supplementary Table 2). Similarly, for the *nonparametric ComBat* corrected dataset, a residual maximum variation of 0.60 (60%) was still detected (Supplementary Table 5), indicating the remaining signal drift across batches for some metabolites. In contrast, the datasets corrected by *ber* and *parametric ComBat* functions presented a similar performance in this study, with a maximum variability associated with the batch < 0.01 (1%) (Supplementary Tables 3–4). These results indicate that both of these functions (i.e., *ber* and *parametric ComBat)* are very efficient in normalizing the signal intensity changes in this large-scale metabolomics dataset.

**Figure 2:**
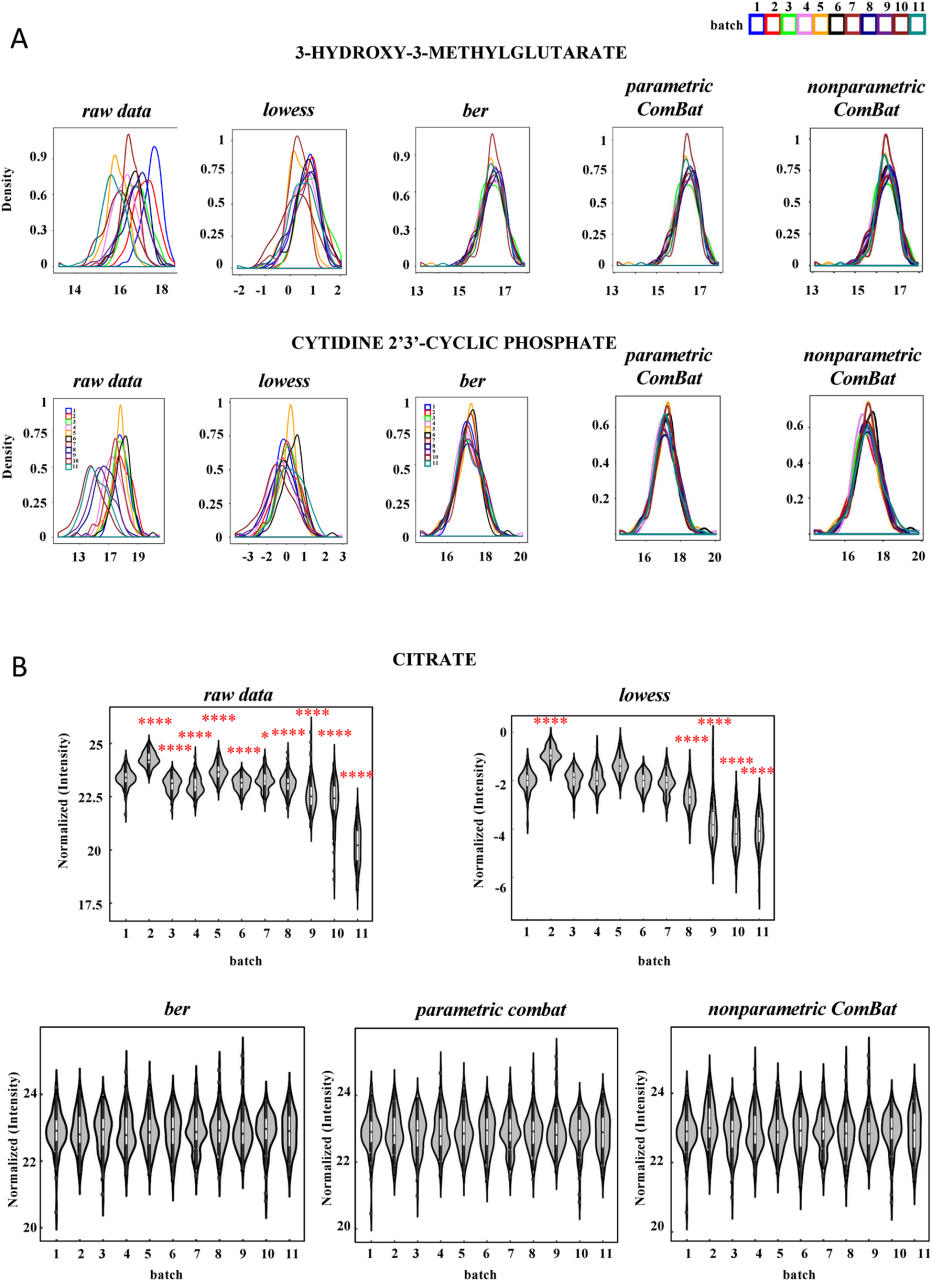
Monitoring of batch effect at feature levels in the LC–MS targeted metabolomics analysis of the SKIPOGH human cross-sectional study with *“dbnorm”*. 1079 plasma Samples were analyzed in 11 analytical batches over a period of 12 months. 239 metabolites were detected. (**A**) Probability density function (*PDF*) plots of 3-Hydroxy-3-methylglutarate and Cytidine 2′,3′-cyclic phosphate across different experimental runs showed the shift in the distribution of signals in *raw* data and its adjustment after data *lowess*-, *ber*-, *parametric ComBat-,* and *nonparametric ComBat-* correction. 3-Hydroxy-3-methylglutarate and Cytidine 2′,3′-cyclic phosphate peaks are detected in positive and negative mode of acquisitions, respectively. (**B**) Violin plots of superposed scatter plots across batches for citrate intensities showed the shift of average distribution across-batches in raw data, which was still present in some batches in *lowess*-adjusted data. This shift is corrected by *ber*, *parametric ComBat,* and *nonparametric ComBat*. Dots correspond to the level of citrate in each sample and the average citrate level in each batch is defined by boxplot. Two-tailed Student’s *t*-test was used to calculate the significant changes of citrate mean level in different batches compared to the batch 1. *, **, *** and **** indicate p-value < 0.05, < 0.01, < 0.001, < 0.0001, respectively.

One peculiar feature of “*dbnorm”* is the possibility of easily visualizing the signal distribution of each feature individually. Probability density function plots (PDF plots) showing the distribution across the batches of the metabolite signal can easily be generated for each metabolite. As an example, PDF plots of 3-hydroxy-3-methylglutarate and 2′,3′-cyclic phosphate 3′-CMP in the SKIPOGH dataset show to what extent each statistical model improves the overlapping signal distribution and reduces the variance scale compared to the raw data (Fig. 2A).

For each metabolite, “*dbnorm*” can also generate violin plots, which further help users to visualize the association of the signal intensity with batch number. Figure 2B shows the clear batch-dependent shift in the signal intensity of citrate, which was observed in the *raw* data and that was still present for some batches in the *lowess*-corrected data. In contrast, in the data corrected with each of the “*dbnorm*” statistical methods, the average intensity of this metabolite remained constant across the batches (Fig. 2B). Another interesting observation in the SKIPOGH dataset was that the use of the statistical models implemented in “*dbnorm”* allowed us to keep some metabolites, such as xanthosine 5′-monophosphate, which was filtered out with *lowess.* Xanthosine 5′-monophosphate is a good example of a low-abundance metabolite, likely present in only one portion of the samples and thus below the limit of detection in the QC samples due to the pooling-dilution effect.

### “*dbnorm*” enables efficient downstream differential analysis

To investigate whether the use of “*dbnorm*” for batch correction allows for coherent data interpretation, we next explored the impact of each type of correction algorithms on the downstream statistical and metabolic pathway analysis in the SKIPOGH study.

**Figure 3:**
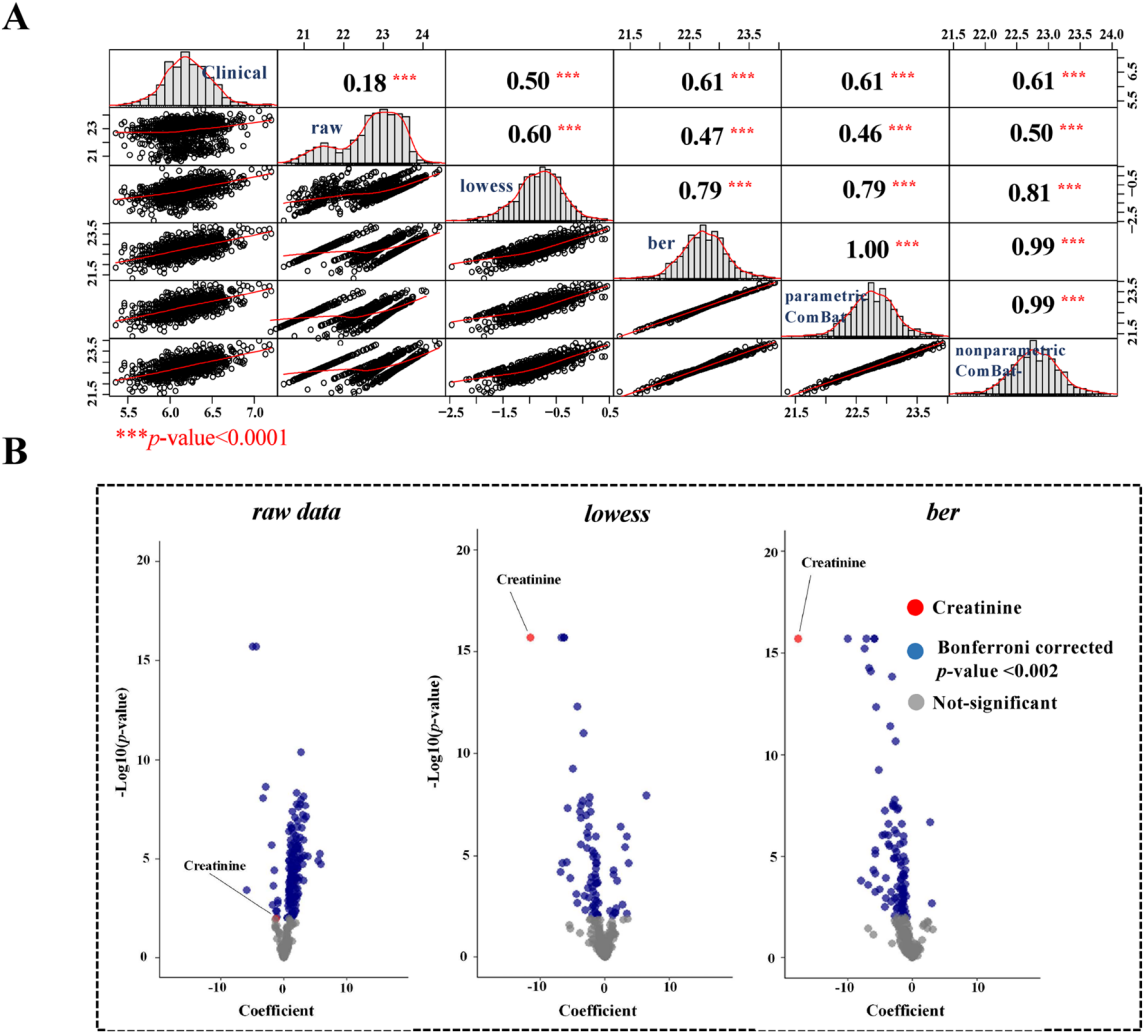
Impact of batch effect correction in LC–MS targeted metabolomics analysis of the SKIPOGH study on downstream differential analysis. Pearson correlation between levels of creatinine, as measured in clinical tests with those measured in LC–MS metabolomics analysis, before or after batch effect correction (**A**). The correlation increases from 0.18 in the *raw* data, to 0.5 in the *lowess*-corrected data, to the maximum of 0.61 in the data corrected via statistical models (*ber*, *parametric* and *nonpararmetic ComBat*). *** indicates *p*-value < 0.001. (**B**) Association of CKD-EPI and metabolite levels fails in detecting significant association with creatinine levels in *raw* data, while this expected association is observed in both *lowess*- and *ber*-corrected datasets. The model is adjusted by age and sex. Bonferroni corrected *p*-value were considered as significant criterion.

All of the subjects of this prospective cohort study were previously phenotyped for kidney functionality and its associated parameters, such as age, sex, and creatinine clearance, as an indicator of renal impairment to estimate the severity of a kidney disease^45^. In addition, the glomerular filtration rate (GFR), a major surrogate of kidney function, was measured on a continuous scale and estimated by the Chronic Kidney Disease Epidemiology Collaboration (CKD-EPI, see the Methods section)^46,47^. We thus took advantage of this clinical information to validate the efficiency of *“dbnorm”* in driving the choice of the best statistical model for batch correction. We first evaluated the correlation between creatinine levels, as determined by the targeted LC–MS/MS metabolomics experiment and those measured in the clinical laboratory with an enzymatic assay. We looked at five datasets: *raw* data, *lowess-*, *ber-* and *parametric*- and *nonparametric ComBat-*corrected. As expected, all of the tested batch effect correction approaches improved the correlation between the clinically measured creatinine levels and those obtained using targeted LC–MS/MS analysis. The correlation coefficient (r) changed from 0.18 (Fisher’s z = 0.18 with a 95% interval level (**CI**), 0.12 to 0.24) observed in the *raw* data, to 0.5 (Fisher’s z = 0.54 with a 95% CI, 0.48 to 0.60) in the *lowess*-corrected data and up to 0.61 (Fisher’s z = 0.71 with a 95% CI, 0.64 to 0.76) for the data corrected by statistical models (Fig. 3A). This observation is indicative of the good performance of *“dbnorm”* statistical models in batch correction.

To further confirm the efficiency of *“dbnorm”*-mediated correction in highlighting relevant biological outcomes, a multiple regression model was applied to the *raw*, *lowess-* and *ber-*corrected data for 934 participants, for which complete metadata were available (see the Methods section). In this model, the CKD-EPI level was considered as an outcome of renal failure, and the creatinine level was considered a predictor. The model was adjusted by sex and age. As presented in Supplementary Table 6A, in the corrected data, creatinine changes across the samples were better predictors of the CKD-EPI changes. In fact, the improved estimation of the known association between CKD-EPI and creatinine^48^ was revealed by the dramatic decrease in the *p*-value and effect size (i.e., the coefficient) in both the *lowess-* and *ber-*corrected data compared to the *raw* data (Fig. 3B, Supplementary Tables 6A–C). In addition, the analysis of variance confirmed that the integration of creatinine levels with sex and age in the regression model improved the prediction across the samples of changes of CKD-EPI when using either *lowess* or *ber*-corrected data (Supplementary Table 7) (see also the Methods section). These observations demonstrate that *ber*, which was scored by *“dbnorm”* as the statistical method of choice for the SKIPOGH dataset, efficiently corrected for batch effects in this dataset, enabling coherent downstream analyses compared to the reference QC method lowess.

## Example analysis 2: batch effect correction in a large-scale untargeted metabolomics dataset

### Assessment and correction of the batch effect with *“dbnorm”*

Untargeted metabolome profiling is mostly employed to increase the chance of identifying unexpected discriminant biological signals, as it allows for the detection of as many metabolite features as possible from diverse chemical classes, without an a priori hypothesis. Due to high levels of noise and redundancy, the use of a robust statistical model for batch effect correction is even more important in untargeted metabolomics datasets. Here, the aim was to evaluate the efficiency of the functions implemented in “*dbnorm*” for batch effect removal in a second totally independent dataset, acquired in a full scan mode in an untargeted high resolution MS.

Briefly, the metabolome profile of two types of adipose tissue, visceral (v-AT) and subcutaneous (sc-AT), was measured in mice fed with a high fat diet (HFD) and/or a control diet (ctrl) for 1 or 8 weeks. Overall, 264 samples were analyzed, including 32 QCs injected every 8 samples. Data acquisition was performed in three separate analytical batches in negative mode, and two batches of continues runs were performed in positive mode. Data processing using XCMS software (https://xcmsonline.scripps.edu/), yielded more than 20 thousand m/z values, sorted and aligned as the features, defining multiparametric metabolic signatures. In particular, 9900 and 11,156 features were obtained for the positive and negative modes, respectively (see the Methods section). Herein, to better show the effect of signal drift across batches, we only present data acquired in the negative polarity.

**Figure 4:**
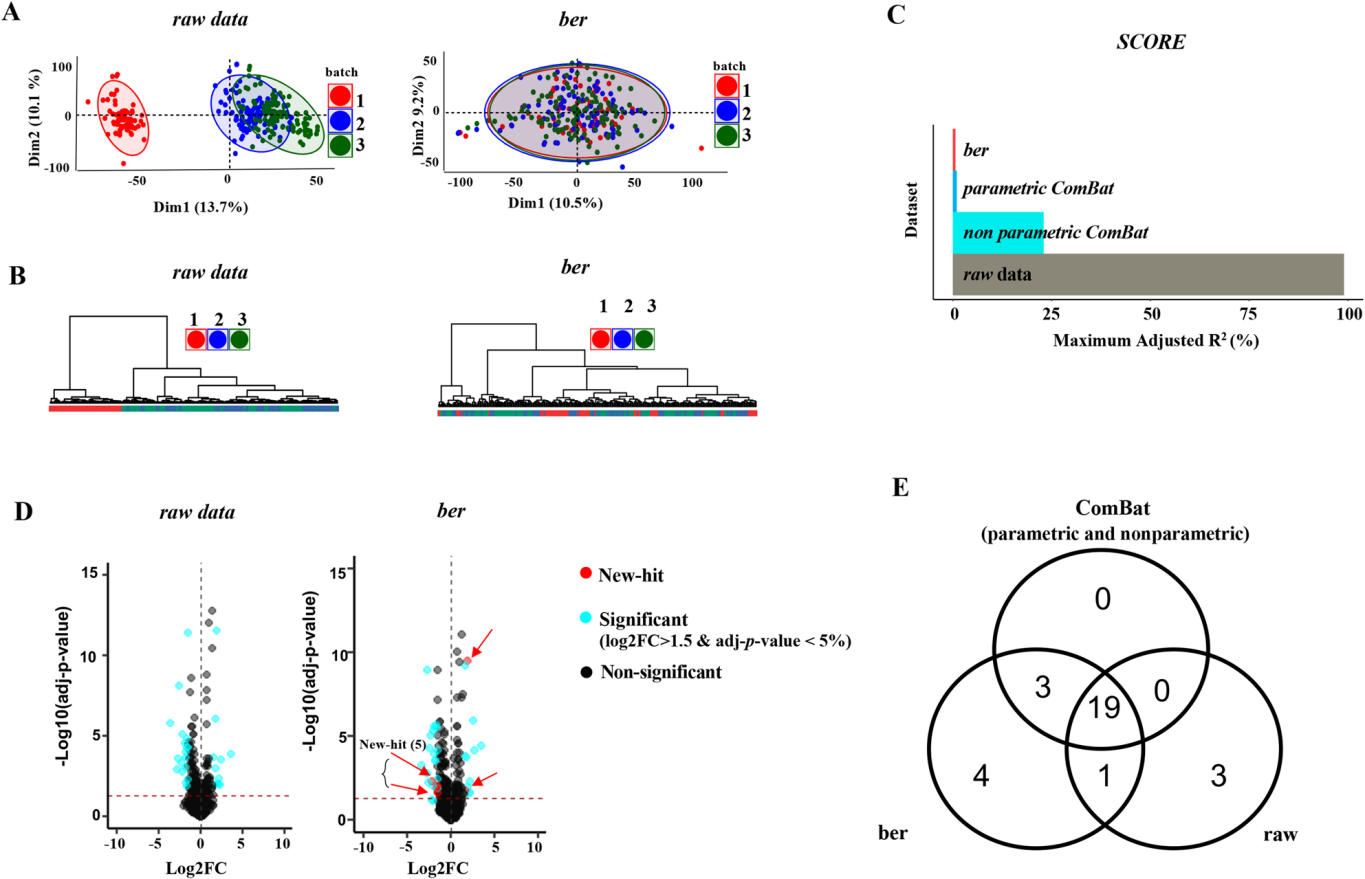
Monitoring of batch effect in LC–MS untargeted metabolomics and its correction with *“dbnorm”*. (**A**) PCA plots of mouse fat tissue metabolome acquired for 264 samples in negative polarity showed sample separation associated with the experimental runs in *raw* data and its correction upon correction using *ber*. (**B**) Dendrogram of *raw* data confirmed clustering of samples linked to batch number, particularly for batch 1. Upon correction using *ber*-function, data points overlapped without any distinct batch driven grouping of samples in dendrograms. The color code indicates the batch number. (**C**) The score plot represents the maximal absolute adj-R^2^ as estimated in raw data and in *ber*-, *nonparametrice ComBat-*, and *parametric ComBat*-corrected data. (**D**) Volcano plots of *raw* and *ber*-corrected data demonstrates the impact of HFD on the list of metabolites whose level significantly changes in response to the treatment. The metabolites appearing as significantly regulated only in *ber*-corrected data are depicted as red dots and arrows. Significant criteria of Log2FC > 1.5 and adj-*p*-value < 0.05 are considered to draw a list of metabolites with differential level. (**E**) Venn diagram showing the number of metabolites significantly altered by 8 weeks of HFD in sc-AT metabolome before and after batch correction through statistical models (*ber*, *par*ametric and *nonparametric* ComBat). Correction with either *parametric* or *nonparametric ComBat* results in the same list of differentially expressed metabolites.

Unsupervised multivariate analysis of 11,156 metabolic profiles (i.e., PCA and Hierarchical Cluster Analysis (HCA)) performed with *“dbnorm”* revealed the presence of a strong batch effect, with clustering of samples according to batches (Fig. 4A,B). In addition, the adjusted coefficient of variation (adj-R^2^) estimated by the regression model indicated that the variability of certain metabolites could be entirely explained by the signal intensity drift across batches (Supplementary Fig. 1B). The statistical models implemented in *“dbnorm”* efficiently removed the batch effect, as suggested by the PCA score plot (Fig. 4A, Supplementary Fig. 1A) and HCA dendrogram (Fig. 4B), with a reduction in variance for the majority of features (potential metabolites) (Supplementary Fig. 1B). Interestingly, the *ber-*corrected data showed the lowest dependency on the batch level, with a consistent effect on all features, as shown by the adj-R^2^ value near zero. The very low negative adj-R^2^ values after *ber* correction are indicative of a very poorly fitted regression model, demonstrating a weak batch effect dependence estimated by the model. The performance of each of the statistical models to correct for batch effect that are implemented in “*dbnorm*” was also visualized by the score plot (Fig. 4C), which confirmed the lowest batch to feature dependency in the *ber*-corrected data.

### “*dbnorm*” enables the discovery of biologically relevant changes

We finally used our untargeted metabolomics dataset to further confirm the efficiency of batch effect correction with “*dbnorm*” to maximize the capture of the true biological differences. To simplify the data interpretation and to rapidly identify trends drawn from the data analysis, we focused on a subset of data. To this end, we investigated the impact of batch effect correction with *“dbnorm”* on the relative changes induced by 8 weeks of HFD in the metabolome of sc-AT. We compared the candidate list obtained from the statistical analysis of *raw* data to that from the data normalized for batch effects using the statistical functions implemented in “*dbnorm*”. The results showed that while the majority of the significant changes were detected in all analyzed datasets, the list of candidate metabolites associated with the HFD treatment was more exhaustive after data adjustment. In particular, as depicted in the volcano plots and in the Venn diagram (Fig. 4D,E), the levels of several metabolites were significantly affected by HFD only after batch effect removal. Interestingly, the majority of these compounds (5 out of 7) belonged to the lipid classes (Supplementary Table 8A), which is consistent with the biological effect of HFD on adipose tissue. This result suggests that, in this dataset, data correction with the best fitting model proposed by “*dbnorm*”, in this case *ber,* enables the detection of biological signals induced by HFD that might be relevant but might be overlooked due to nonbiological variation between samples.

To further explore the relevance of the additional biological signals highlighted after data correction with *“dbnorm”*, we next annotated the metabolites that were significantly influenced by HFD to functional pathways by using metabolite set enrichment analysis (MSEA). We focused only on the *ber*-corrected data since the list of significant metabolites obtained with these data included all of those defined by *ComBat*-corrected data. Interestingly, as shown in Supplementary Fig. 2, some lipid metabolism pathways, such as “alpha-Linolenic acid metabolism” and “Fatty acid biosynthesis”, were enriched, as dysregulated functions, only using the list of the differential metabolite derived from *ber*-corrected data (Supplementary Fig. 2). Of note, these two pathways were previously shown to be influenced by HFD in adipose tissue^49,50^. Few metabolites belonging to these two pathways were also identified as significant in the *raw* data (Supplementary Table 8A), but their numbers were not sufficient to trigger an enrichment of the corresponding pathways (Supplementary Table 8B).

Therefore, batch effect removal using the *ber* function implemented in *“dbnorm”* improved the data structure and enabled us to better capture relevant biological signals in our study in a mouse model of obesity. Collectively, our demonstrative analyses strongly indicated that *“dbnorm”* is an efficient tool in helping users analyze the structure of their data. Moreover, it provides choices among several statistical models that showed good performance in metabolomic datasets, as demonstrated by their comparison with a reference QC sample-based method for data correction.

## Discussion

Metabolic profiling offers holistic determination of intermediate and end products of metabolism whose deviation from normal levels might provide important information on the dysregulation of metabolic pathways in disease conditions. Such changes can be caused by genetic disorders, environmental factors, drug treatment, etc. This information could help improve disease diagnosis, prognosis and treatment choices. However, population-based studies need efficient experimental design and data preprocessing to generate comparable sample sets across batches.

Our study, in agreement with previous reports, supports the necessity of data cleaning to remove unwanted technical variation, which helps improve the detection of biological mechanisms underlying a medical state. Rather than highlighting new biological findings associated with phenotypes of the two sets of samples that we analyzed here, our study aimed to provide clear-cut evidence that the choice of an appropriate normalization model is crucial to remove nonbiological variation to better determine the true biological effect of clinical conditions and diet treatments. We provide “*dbnorm*” as an efficient and user-friendly tool for the removal of drift across batches.

Our results show how *“dbnorm”* helps users diagnose the presence of analytical drift through the generation of a panel of graphs depicting PCA and distance clustering analysis to assess data structure at the sample level, and also scatter, violin and density plots to explore data at the feature level. Moreover, “*dbnorm*” provides choices among several state-of-the-art statistical models, including *ComBat* (both *parametric* and *nonparametric* versions) and *ber* (including its bagging version), whose performance in batch effect correction can be easily evaluated through the generation of the same panel of graphs and through the estimation of an adj. R^2^ score, which notifies the user about the percentage of variance in the corresponding datapoint associated with the batch level. Our example analyses clearly show that the statistical models implemented in “*dbnorm*” can perform an accurate correction of batch correction, as demonstrated by their comparison with a reference QC sample-based method for data correction.

Periodic injection of QC samples is one of the most commonly used methodologies in the metabolomics community and is exploited by QC sample-based correction models. However, this methodology has the limitation of being dependent on the availability of QCs truly representative of all of the study samples, whose adequate preparation is not always possible, particularly in large-scale studies. If surrogate QC samples are used, QC-based normalization methods might fail to remove batch effects homogenously for all of the features characterizing the multiparametric metabolic profile. Our results confirm some limits of such correction methods. For instance, we showed that in the human dataset that we analyze, citrate levels remained highly associated to the batch order in the *lowess*-adjusted data, which might generate bias in data interpretation. In addition, the analysis with a QC sample-based correction model is restricted only to the metabolites that are detected in both the QC and the study samples, thus potentially impairing the discovery of novel biological hits in a medical condition. As another example, xanthosine 5′-monophosphate was only detected in the study samples but not in the QCs and was therefore discarded a priori by the *lowess* model. In our human population dataset, while variability linked to batches is still present for some metabolites by using QC sample-based *lowess* correction model, all of the statistical models implemented in the “*dbnorm*” package present a higher performance on the overall correction of the signal drift across batches, with *parametric ComBat* and *ber* showing the best score in reducing the association between a metabolite feature and the batch level. We cannot exclude that other datasets with a different data structure might be more effectively adjusted by *nonparametric ComBat* and QCsample-based models.

Our results clearly demonstrate the substantive impact of data adjustment for analytical heterogeneity on the prediction of clinical outcomes. In the human study, data normalization triggered an increased association between eGFR (the outcome measured via the CKD-EPI formula) and creatinine, thus highlighting a pattern that was not detected in the *raw* data. This result indicates that batch effect correction performed on our dataset favors the detection of biologically relevant differences. Of note, the *ber* and *ComBat* functions have both been developed to avoid overfitting of the data in the case of a small sample set in each batch.

In the mouse-model experiment, the statistical models compensating for across-batch signal drift drastically decreased the high variability associated with the batch level in the *raw* dataset to an almost zero level in the corrected datasets, with a more consistent removal observed when employing the *parametric ComBat* model and *ber* model. Data correction resulted in a slightly distinct list of differential features associated with HFD treatment, with similar candidates given by functions such as *ber*, *parametric ComBat* and *nonparametric ComBat*. Although the list generated by using the *ber* function added up to only a few metabolite candidates, these metabolites belong to different lipid classes that, in fact, are known to be controlled by HFD. As a consequence, this difference increased the enrichment of two biological pathways that are known to be affected by HFD treatment, thus improving the biological significance of the results.

In conclusion, “*dbnorm*” assists users in visualizing the structure of large metabolomic datasets before and after correction via the implemented statistical methods, not only from the perspective of samples analyzed in the entire experiment, but also from that of the metabolic features detected in the study samples. Importantly, “*dbnorm*” can also be used for metabolomic studies lacking appropriate QC samples. In the future, “*dbnorm*” and its application could be extended to other high-throughput techniques.

## Methods

### Package

“*dbnorm*” and its functions are explained in detail in the Package Manual. Briefly, it includes distinct functions for pre-processing of data and estimation of missing values, conventional functions for batch effect correction based on statistical models, as well as functions using advanced statistical tools to generate several diagnosis plots to inform users about their data structure. The “*dbnorm*” package includes statistical tools which allows user to inspect the structure and quality of multidimensional datasets of large metabolomics datasets at both macroscopic and microscopic scale, namely at the sample batch level and metabolic feature level, respectively. This package is publicly available at https://github.com/NBDZ/dbnorm .

Batch correction models implemented in the “*dbnorm*” are adapted from microarray analysis, namely, *ber* statistical model, including its *bagging* variant (ber: https://cran.r-project.org), and *ComBat* in both settings of *parametric* and *nonparametric* from sva package in R (sva; https://bioconductor.org). In brief, *ComBat* uses EB method to remove location (mean) and scale (variance) of batch effect.

In contrast, *ber* function uses linear regression at two stages to estimate location and /or scales parameters^43^.

The *bagging* variant of *ber* model uses aggregated prodictors with better accuracy that are obtained from bootstrapped predictors. In “dbnorm” we considered a bootstrap sample size of n = 150 and a partial bagging procedure.

### Human study

SKIPOGH (Swiss Kidney Project on Genes in Hypertension) is a family-based multi-center population-based study exploring the role of genes and kidney haemodynamics in blood pressure (BP) regulation and kidney function. Method and population are described in detail elsewhere^51–53^. Briefly, in this study 1079 plasma samples from SKIPOGH population have been considered. For all samples information for gender and age were available, while CKD-EPI levels were only available for 934 samples. The SKIPOGH study was approved by the institutional ethical committees of the three participating university hospitals (Lausanne, Geneva, Bern) and was performed in accordance with Swiss guidelines and regulations. All study participants provided written informed consent.

### Animal experimentation

All animal experiments and procedures were approved by the Swiss Veterinary Office (VD-2942.b. and VD-3378), were performed in accordance with Swiss guidelines and regulations (OPAn) and were carried out in compliance with the ARRIVE guidelines for using animal in studies. C57/BL6 male mice were purchased from Janvier Labs and housed 5 per cage in the animal facility of Centre for Integrative Genomics, University of Lausanne.

Four-week old mice were fed for two weeks with a 10% in fat chow diet (D12450J, Research Diet). At 6 weeks of age they were either shifted to a high-fat diet (HFD) containing 60% fat (D12492, Research Diet) or kept on a control diet for 1 or 8 weeks. Random blocking was used. Efficiency of the diet-induced obesity was followed by regular measurements of weight^54^. All animals were kept in a 12:12 h light:dark cycle with water and food ad libitum. All the mice were sacrificed by CO_2_ between ZT2 and ZT5. In this study sc-AT refers to inguinal subcutaneous adipose tissue in mice.

### Metabolomics

Targeted metabolomics analysis conducted on the plasma samples of the human prospective cohort study. Metabolites were extracted from 100 µL samples using a methanol-ethanol solvent mixture in a 1:1 ratio. After protein precipitation, supernatant was evaporated to dryness and finally re-suspended to 100µL H_2_O 10% MeOH. The samples were analyzed by LC-MRM/MS on a hybrid triple quadrupole-linear ion trap QqQ_LIT_ (Qtrap 5500, Sciex) hyphenated to a LC Dionex Ultimate 3000 (Dionex, Thermo Scientific). Analysis were performed in positive and negative electrospray ionization using a TurboV ion source. The MRM/MS method included 299 and 284 transitions in positive and negative mode respectively, corresponding to 583 endogenous metabolites. The Mass Spectrometry Metabolite Library (Sigma Aldrich) was used as reference material for the standard metabolites.

The chromatographic separation was performed on a column Kinetex C18 (100 × 2.1 mm, 2.6 µm). The mobile phases were constituted by A: H2O with 0.1% FA and B: Acetonitrile (ACN) with 0.1% formic acid (FA) for the positive mode. In the negative mode, the mobile phases were constituted by A: ammonium fluoride 0.5 mM in H_2_O and B: ammonium fluoride 0.5 mM in ACN.

The linear gradient program was 0–1.5 min 2%B, 1.5–15 min up to 98%B, 15–17 min held at 98% B, 17.5 min down to 2%B at a flow rate of 250 µL/min.

Total 1079 different plasma samples were analyzed in 11 batches over 12 months. 135 and 156 QCs were also periodically injected between sample runs in the positive- and negative- modes respectively. Surrogated QCs were considered in this study to prevent repeated thawing-refreezing cycles.

The MS instrument was controlled by Analyst software v.1.6.2 (AB Sciex). Peak integration was performed with MultiQuant software v.3.0 (AB Sciex). The integration algorithm was MQ4 with a Gaussian smoothing of a half-width equal to 1.5 points.

On the other hand, to obtain the fat metabolome profile of mice, metabolites were extracted from 10–20 mg of fat depots either sc-AT or v-AT using 400 µL of mix organic solvent comprising EtOH: MeOH: H_2_O in the proportion of 2:2:1 to remove protein efficiently as well as to extract polar and semi-polar metabolites successfully. All the samples were then vortexed mixed for 30 s, incubated for 10 min at 4 °C and centrifuged for 10 min at 14,000 rpm and 4 °C. The supernatants were removed and evaporated to dryness using speed vacuum concentrator (SpeedVac) and stored at -80 °C until analysis. QCs were prepared by pooling all the tissue integrated in the study. Extraction was done using similar protocol use for the samples. Supernatant were aliquots in 34 tubes considering similar quantity. Then they were treated like samples.

Untargeted metabolomics approach applied in this study has been described in our previous papers^55,56^. Briefly, untargeted metabolomics was performed using UPLC (Dionex,Thermo Fisher Scientific) hyphenated with HRMS QExactive plus (Thermo ScientificTM Q ExactiveTM). Metabolome profile of fat tissue (i.e. v-AT and sc-AT) was obtained for 264 samples including 32 QCs and 232 adipose tissue Samples were injected in two batch blocks in a random order. QCs were injected within each batch run regularly (every 7 to 8 samples), to assess data quality. HRMS was interfaced with an electrospray ionization (ESI) source operated in both negative (NEG) and positive (POS) polarities. Data acquisition in POS mode has been finalized in two days of continues run, while NEG mode was completed in three separated analytical runs. ESI source was optimized using sheath gas flow rate 40, auxiliary gas flow rate 10, capillary temperature 320 °C, S-lens RF 50 and auxiliary gas heater temperature 300 °C. All the ESI parameters were identical in both ionization modes except the sweep voltage which was selected to have proper spray current (for POS 3.3 kV and for NEG 2.9 kV). Moreover, lock mass was considered with respect to the acquisition mode which permit real-time recalibration by correcting m/z shifts compensating for the instrumental drift. UPLC was performed on a C18 Kinetex, 2.6 μm, 50 mm × 2.1 mm I.D. column (Phenomenex, PA) in Reverse Phase chromatography. Mobile phase was composed of A = 0.1% Formic acid in H2O and B = 0.1% Formic acid in MeOH for both positive and negative ionization modes. Elution was carried out in gradient condition and mobile phase composition changed from 98% A (0–6 min) to 100% B (6.1–9 min) and stabilized for 4 min for column re-equilibrating likewise to keep analysis reproducibility. The overall run time was 13 min. Flow rate was set at 0.3 mL/min and the sample injection volume were 2.5μL counting for half loop portion. Identity of interested features confirmed mainly using a targeted selected ion monitoring (tSIM) data dependent MS/MS acquisition method (tSIM/ddMS/MS). In tSIM/ddMS/MS, the precursors enlisted in the inclusion list were selected in the quadrupole with 2 Da isolation width, followed by a ddMS/MS scan with the similar isolation window. The number of precursor scans for co-eluting analytes (MSX) was set at 1. The precursor ions were selected using loop count = 5, MSX = 1 and MS/MS spectra acquired simultaneously for 5 ions within the isolation windows at each scan. Resolutions were set at 70,000 and 17,000 for tSIM and ddMS/MS respectively. The AGC targets for tSIM were set at 2 × 105 with the maximum inject time (IT) of 150 ms and for ddMS/MS at 1 × 105 with IT = 100 ms. MS/MS spectra were generated when the signal reaches the threshold of 1%.

Yet, some interesting metabolic features were notified as semi-identified, that means they are identified as a potential meknown metabolite based on their accurate mass, real peak shape and retention time.

*Raw* data were then transformed to mzXML format using MSConvert (Proteo Wizard 3.07155) and pre-processed for peak peaking, chromatogram alignment and isotope annotation using open access XCMS online (https://xcmsonline.scripps.edu). XCMS runs on UPLC-QExactive parameters by setting peak detection on Centwave.

### Chemometrics and Pathway analysis

For analysis, *raw* data generated in the targeted metabolomics were log2-transformed for each metabolite. Further normalization for across-batch signal drift was done using either QC-based model specifically *lowess*-model from open access web page (http://prime.psc.riken.jp/Metabolomics_Software/LOWESS-Normalization/) or non-QC based algorithms using “*dbnorm*” package. Notably, data for each mode of acquisition was treated separately for batch effect removal through either of QC-based or non-QC-based model, and then merged for visual check and downstream differential analysis. A total of 239 different plasma metabolites detected in a human prospective cohort study among which XANTHOSINE 5′-MONOPHOSPHATE was missing in QCs analyzed in positive modes.

Untargeted metabolomics data subjected to batch effect was also treated for normalization of a cross- batch signal drift using statistical methods implemented in the “*dbnorm*” package, which is also used for visualization of the outcome. In fact, in this study, the major focus is given to negative polarity in which data acquisition were completed in three separated analytical runs and thereby typically subjected to batch effects.

Metabolome signature in either of study, human study and animal experiment, was obtained by linear logistic regression model using lm (R *Stats* Package) and *limma* package (https://bioconductor.org), respectively.

In the human cohort study, even though metabolomics analysis and data processing has been performed on all 1079 samples, evaluation of differential biological effect performed on 943 participants for which information on CKD-EPI level were available. GFR is estimated by an equation developed by the Chronic Kidney Disease Epidemiology Collaboration (CKD-EPI) used as an outcome of renal impairment. CKD-EPI as estimator of kidney function calculated by gender and stratified by creatinine is assessed via formula presented in the reference^57^.

Pearson’s r is calculated to rigorously estimate relationship between creatinine level measured in clinical routine and by MS. CIs at α < 0.05 (i.e. at 95% level) were produced upon transformation of pearson’s r to fisher’s z.

Subsequently, the association between CKD-EPI and plasma metabolome were investigated by multiple regression testing adjusted for age and sex. Bonferroni corrected p-value was considered as the significant criterion for the metabolite selection.

Then, volcano plot was used to visualize significance and the magnitude of changes in metabolites associated with CKD-EPI level.

In mouse model of obesity, we mainly focused on sc-AT metabolome signature driven by HFD after 8 weeks treatment, in comparison with their control counterpart. Linear regression model was used and significant criteria for metabolite selection were set at Log2FC > 1.5 and benjamini and bochberg (BH) adjusted *p*-value < 0.05.

Subsequently, significant list was further searched and filtered against a Human Metabolome Database (HMDB; http://www.hmdb.ca) to keep only potential hits that were ultimately either partially confirmed based on peak shape and retention time denoted as “semi” or fully confirmed based on MS/MS spectra.

Differential list associated with 8 weeks of HFD treatment was then subjected to over-representation analysis (ORA) using web interface of ConsensusPathDB ( http://consensuspathdb.org/) to pinpoint biochemical pathways that are dysregulated and may have a causative relationship to the phenotype. The list of identifiers was mapped to predefined KEGG pathways database enlisted by 4289 compound IDs. As defined by software, “*p*-value is calculated according to the hypergeometric test based on the number of physical entities present in both the predefined set and user-specified list of physical entities” (http://cpdb.molgen.mpg.de/). Result includes q-value which is in fact the *p*-values corrected for multiple testing using the false discovery rate. The selection criteria for a significantly dysregulated biological function was set to at least two metabolites representing the biological pathways with q-value < 0.05. Significant pathways were visualized ed using bar plot. In this plot, primary y-axis notifies Metabolite’s coverage, calculated as relative number of input metabolite define a pathway to a whole metabolite set of a pathway. Secondary y-axis presents q-value defined for each pathway in negative log2 scale.

## Supplementary Information

Supplementary Information 1.

Supplementary Information 2.
