# Supplementary material for: DBnorm as an R package for the comparison and selection of appropriate statistical methods for batch effect correction in metabolomic studies
Source: Sci Rep. 2021 Mar 11;11:5657. doi: 10.1038/s41598-021-84824-3 (PMC7952378; doi:10.1038/s41598-021-84824-3)
Supplement: Supplementary file 1 — Supplementary Information 1. [file 41598_2021_84824_MOESM1_ESM.pdf]

DBnorm as an R package for the comparison and selection of appropriate statistical methods for batch effect correction in metabolomic studies

Nasim Bararpour[1,2], Federica Gilardi[1,2], Cristian Carmeli[3,4], Jonathan Sidibe[1], Julijana Ivanisevic[5], Tiziana Caputo[6], Marc Augsburger[1], Silke Grabherr[7], Béatrice Desvergne[6], Nicolas Guex[8], Murielle Bochud[3], Aurelien Thomas[1,2] *

[1]Unit of Forensic Toxicology and Chemistry, CURML, Lausanne University Hospital-Geneva University Hospitals, Switzerland.

[2]Faculty Unit of Toxicology, CURML, Lausanne University Hospital, Faculty of Biology and Medicine, University of Lausanne, Switzerland.

[3]Center for Primary Care and Public Health (Unisanté), University of Lausanne, Lausanne, Switzerland

[4]Population Health Laboratory, Department of Medicine and Public Health, University of Fribourg, Fribourg, Switzerland

[5]Unit of Metabolomics, Department of Biology and Medicine, University of Lausanne, Switzerland

[6]Center for Integrative Genomics, University of Lausanne, Lausanne, Switzerland

[7]CURML, Lausanne University Hospital-Geneva University Hospitals, Switzerland

[8]BioInformatics Competence Center, University of Lausanne, Lausanne, Switzerland

***Corresponding Author:**

Aurélien Thomas: aurelien.thomas@chuv.ch

Table of content

1. **Supplementary Figures 1-2** **page 3**
2. ***"dbnorm"* manual** **page 5**
3. **Supplementary Tables 1-8 are in the supplementary .xlxs fil**e:
   Bararpour et al_Supplementary Tables 1_to_8.xlxs

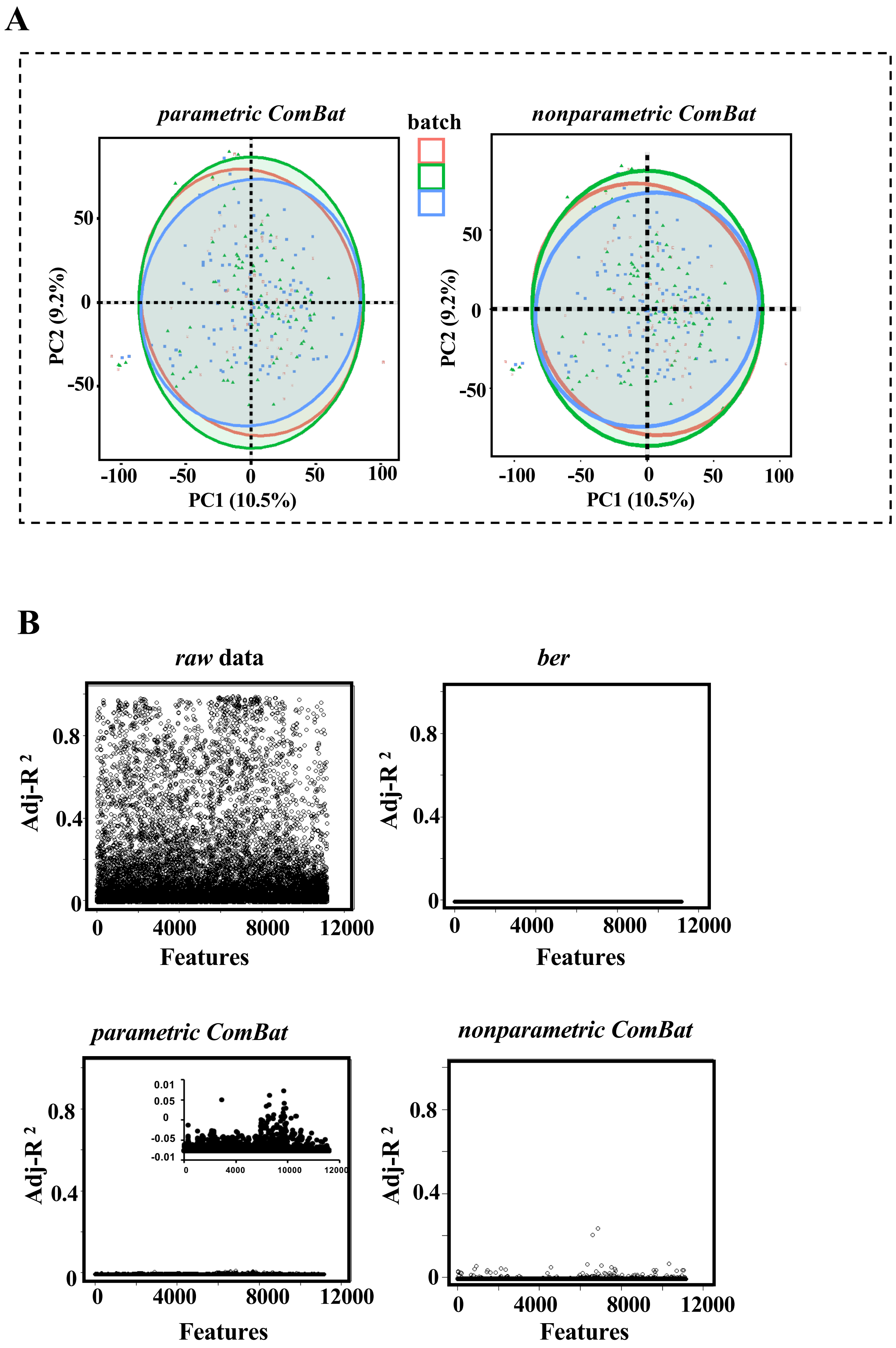

Supplementary Figure 1. Graphical check for the performance of statistical models in batch effect removal in LC-MS untargeted metabolomics analysis of 264 adipose tissue samples. **(A)** Principal Component Analysis (PCA) score plot of parametric and nonparametric *ComBat*-corrected data do not show any separated clusters of samples along the experimental runs. **(B)** Adjusted Coefficient of Determination (Adjusted R-squared), as estimated by regression model, shows that the variability dependent to the batch level in raw data is about 100%, while a profound decrease to almost zero is detected in the *ber*-corrected dataset. Reduced association between feature variability and batch level is also observed in parametric *ComBat*-corrected dataset, as visualized in both auto-scaled and adjusted scaled graph, and in nonparametric *ComBat*-corrected dataset. The negative Adjusted R-squared values detected here are usually determinant of poor fitted model to assess variability.

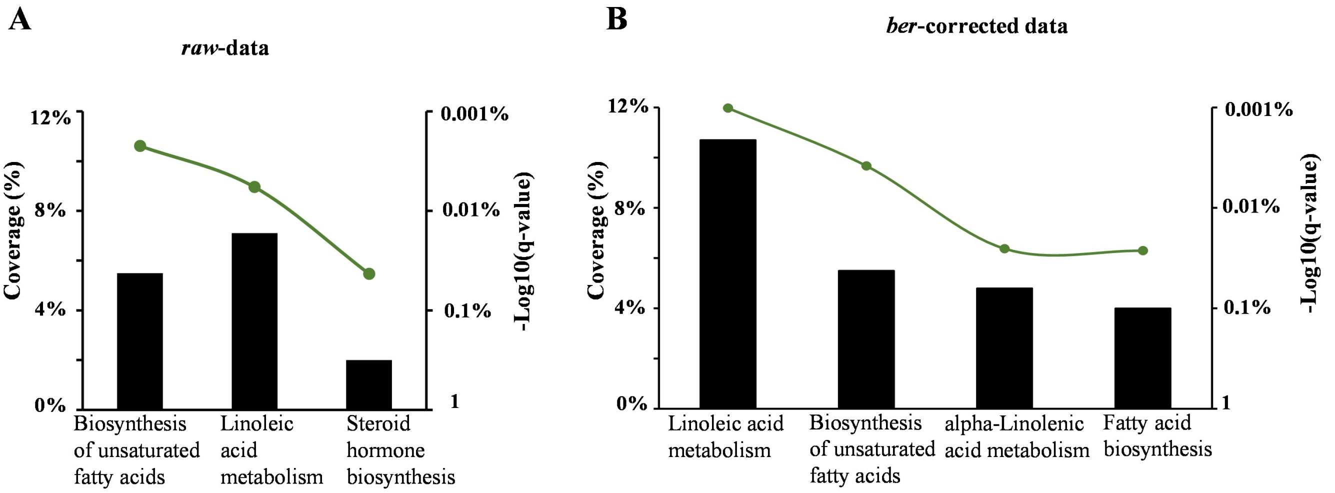

Supplementary Figure 2. Pathway enrichment analysis of the differential metabolites in sc-AT metabolome in response to 8 weeks of HFD. Biological pathways significantly regulated by HFD were obtained using *raw* data **(A)** or the *ber*-corrected data **(B)** as inputs. A threshold of at least two candidates to define a pathway with a *q*-value<0.05 was applied. The graphs show the metabolite's coverage for the list of pathways on the primary y-axis, while secondary y-axis notifies the significant level of the pathway, reported as q-value (adj-*p*-value**).**

Package ‘dbnorm’

November 22, 2020

Type Package

Title Drift Across-Batches Normalization and Visualization

Description dbnorm includes several functions applicable in a large-scale Metabolomics analysis as well as other big data. Notably, it includes distinct functions for processing of data and estimation of missing values, functions for batch effect correction based on using several statistical models together with various graphical checks and visualize inference. By evaluating model performances from multiple perspectives, sample batch and metabolic features and by comprehensive visualization of data structure, “dbnorm” enabled inclusive comparison among the integrated methods.

Version 0.2.2

Maintainer Nasim Bararpour <nasimbararpour@gmail.com>

Encoding UTF-8

License GPL-3

Depends R (>= 3.5)

URL https://github.com/NBDZ/dbnorm

Imports sva ,ber(<= 4.0), NormalizeMets(<= 0.25),factoextra, ggplot2

Suggests
AUC,base,Biobase,BiocParallel,devtools,DiffCorr,e1071,edgeR,fs,GGally,ggfortify,graphics,
grDevices,installr,knitr,limma,MASS,MetNorm,pcaMethods,plotly,processx,RGtk2,
rmarkdown,stats,statTarget,tibble,usethis,utils

LazyData yes

RoxygenNote 7.1.1

R topics documented:

dbnormBagging 2
dbnormBer 3
dbnormNPcom 4
dbnormPcom 5
dbnormSCORE 6
emvd 7
emvf 8
hclustdbnorm 9
ProfPlotBagging 10
ProfPlotber 11

ProfPlotComNPara 12
ProfPlotComPara 13
ProfPlotraw 14
Visodbnorm 15

Index **16**

`dbnormBagging` *Drift Across Batch Normalization applying bagging model and visualization*

Description

It is a function in `dbnorm`, a package in R. This function allows users to effectively remove variance associated with batch from data by applying L/S method and partial bagging with Bootstrap samples of size n=150, as explain by M.Giordan. In fact, in *dbnormBer*, we included various types of graphical check for visualization of data point, sample-wise and feature-wise. Considering a single function as a correction algorithm, we aimed to fasten computational processing of big data.
Notably, using this function users applied unsupervised learning algorithm to visualize the most variance explained by the two first components in the different set of samples analyzed in the entire experiment in the raw and corrected data. In parallel, linear association of feature (variable) and batch level has been estimated and visualized by a correlation plot. In fact, estimated *Adjusted- R2* is considered to define the percentage of variance in a dependent variable estimated by independent variable (batch) in a original data (Raw data) and in corrected data. Besides, for quick notification about the performance of the applied model a maximum variability detected in either of datasets is reported as a score. This score notifies the consistency of model performance for all detected features (variables).

Usage

```
dbnormBagging(m)
```

Arguments

`m`	A data frame in which rows define the independent experiments (samples) and columns the features (variables), with the batch levels in the first column.

Details

Zero and NA values are not allowed. Optionally missing value can be imputed by the functions such as `emvf` and /or `emvd` implemented in the `'dbnorm'` package. Input must be normalized and transformed prior.

Value

Several graphs compiled into a **PDF** file are a *PCA* score plot, *Scree* plot and a *correlation* plot estimated for raw and corrected data. Also, the *RLA* plot for each dataset visualized in the **Viewer** panel in the **rstudio** console.
Files saved as **csv** in the working directory are a dataset corrected by the applied model. Also, a two column matrix for Adjusted R-Square raw and corrected dataset and a table summarizing the maximum score.

References

M.Giordan (2013) < DOI:10.1007/s12561-013-9081-1> *https://link.springer.com/article/10.1007/s12561-013-9081-1*

Examples

```
## Not run:
batch<- rep(gl(5,10,labels = c(1:5)),1)
y<- matrix(rnorm(5000),nrow=50)
m<-data.frame(batch,y)
dbnormBer(m)
## End(Not run)
```

---

dbnormBer *Drift Across Batch Normalization via ber- model and visualization*

Description

It is a function in dbnorm, a package in R. This function allows users to effectively remove variance associated with batch from data by applying L/S method as explain by M.Giordan. In fact, in *dbnormBer*, we included various types of graphical check for visualization of data point, sample-wise and feature-wise. Considering a single function as a correction algorithm, we aimed to fasten computational processing of big data.
Notably, using this function users applied unsupervised learning algorithm to visualize the most variance explained by the two first components in the different set of samples analyzed in the entire experiment in the raw and corrected data. In parallel, linear association of feature (variable) and batch level has been estimated and visualized by a correlation plot. In fact, estimated *Adjusted- R2* is considered to define the percentage of variance in a dependent variable estimated by independent variable (batch) in a original data (Raw data) and in corrected data. Besides, for quick notification about the performance of the applied model a maximum variability detected in either of datasets is reported as a score. This score notifies the consistency of model performance for all detected features (variables).

Usage

```
dbnormBer(m)
```

Arguments

m	A data frame in which rows define the independent experiments (samples) and columns the features (variables), with the batch levels in the first column.

Details

Zero and NA values are not allowed. Optionally missing value can be imputed by the functions such as emvf and /or emvd implemented in the 'dbnorm' package. Input must be normalized and transformed prior.

Value

Several graphs compiled into a **PDF** file are a *PCA* score plot, *Scree* plot and a *correlation* plot estimated for raw and corrected data. Also, the *RLA* plot for each dataset visualized in the **Viewer** panel in the **rstudio** console.
Files saved as **csv** in the working directory are a dataset corrected by the applied model. Also, a two column matrix for Adjusted R-Square raw and corrected dataset and a table summarizing the maximum score.

References

M.Giordan (2013) < DOI:10.1007/s12561-013-9081-1> *https://link.springer.com/article/10.1007/s12561-013-9081-1*

Examples

```
## Not run:
batch<- rep(gl(5,10,labels = c(1:5)),1)
y<- matrix(rnorm(5000),nrow=50)
m<-data.frame(batch,y)
dbnormBer(m)
## End(Not run)
```

`dbnormNPcom` *Drift Across Batch Normalization via Parametric- ComBat model and visualization*

Description

It is a function in `dbnorm`, a package in R. This function allows you adjust the data for signal drift across multiple batches or batch effect using non-parametric ComBat methods (see "ComBat" in "sva", a package in bioconductor). Including single method in *dbnormNPcom*, we aimed to fasten the computational processing of big data. This function includes various types of graphical check for visualization of data point, sample-wise and feature-wise.
Notably, using this function users applied unsupervised learning algorithm to visualize the most variance explained by the two first components in the different set of samples analyzed in the entire experiment in the raw and corrected data. In parallel, linear association of feature (variable) and batch level has been estimated and visualized by a correlation plot. In fact, estimated *Adjusted- R2* is considered to define the percentage of variance in a dependent variable estimated by independent variable (batch) in a original data (Raw data) and in corrected data. Besides, for quick notification about the performance of the applied model a maximum variability detected in either of datasets is reported as a score. This score notifies the consistency of model performance for all detected features (variables).
#'

Usage

```
dbnormNPcom(m)
```

Arguments

`m`	A data frame in which rows define the independent experiments (samples) and columns the features (variables), with the batch in the first column.

Details

Zero and NA values are not allowed. Optionally missing value can be imputed by the functions such as `emvf` and /or `emvd`, functions implemented in `'dbnorm'` package. Input must be normalized and transformed prior.

Value

Several graphs compiled into a **PDF** file are a *PCA* score plot, *Scree* plot and a *correlation* plot for raw and corrected dataset. Also, the *RLA* plot for each dataset visualized in the **Viewer** panel in the **rstudio** console.
Files saved as **csv** in the working directory are a dataset corrected by the applied model. Also, a two column matrix for Adjusted R-Square raw and corrected dataset and a table summarizing the maximum score.

References

Johnson et al.(2007) *http://www.ncbi.nlm.nih.gov/pubmed/16632515*
Leek et al. (2012) *https://www.ncbi.nlm.nih.gov/pmc/articles/PMC3307112/*

Examples

```
## Not run:
batch<- rep(gl(5,10,labels = c(1:5)),1)
y<- matrix(rnorm(5000),nrow=50)
m<-data.frame(batch,y)
dbnormNPcom(m)
## End(Not run)
```

`dbnormPcom` *Drift Across Batch Normalization via Parametric- ComBat model and visualization*

Description

It is a function in `dbnorm`, a package in R. This function allows you adjust the data for signal drift across multiple batches or batch effect applying emphEmpirical Bayes method (see "ComBat" in "sva", a package in bioconductor). Including single method in *dbnormPcom*, we aimed to fasten the computational processing of big data. This function includes various types of graphical check for visualization of data point, sample-wise and feature-wise.
Notably, using this function users applied unsupervised learning algorithm to visualize the most variance explained by the two first components in the different set of samples analyzed in the entire experiment in the raw and corrected data. In parallel, linear association of feature (variable) and batch level has been estimated and visualized by a correlation plot. In fact, estimated *Adjusted- R2* is considered to define the percentage of variance in a dependent variable estimated by independent variable (batch) in a original data (Raw data) and in corrected data. Besides, for quick notification about the performance of the applied model a maximum variability detected in either of datasets is reported as a score. This score notifies the consistency of model performance for all detected features (variables).

Usage

```
dbnormPcom(m)
```

Arguments

m	A data frame in which rows define the independent experiments (samples) and columns the features (variables), with the batch levels in the first column.

Details

Zero and NA values are not allowed. Optionally missing value can be imputed by the functions such as emvf and /or emvd implemented in dbnorm package. Input must be normalized and transformed prior.

Value

Several graphs compiled into a **PDF** file are a *PCA* score plot, *Scree* plot and a *correlation* plot for raw and corrected data. Also, the *RLA* plots for each dataset visualized in the **Viewer** panel in the **rstudio** console.
Files saved as **csv** in the working directory are a dataset corrected by the applied model. Also, a two column matrix for Adjusted R-Square raw and corrected dataset and a table summarizing the maximum score.

References

Johnson et al., (2007) < DOI:10.1093/biostatistics/kxj037 > *http://www.ncbi.nlm.nih.gov/pubmed/16632515*
Leek et al., (2012) < DOI:10.1093/bioinformatics/bts034> *https://www.ncbi.nlm.nih.gov/pmc/articles/PMC3307112/*

Examples

```
## Not run:
batch<- rep(gl(5,10,labels = c(1:5)),1)
y<- matrix(rnorm(5000),nrow=50)
m<-data.frame(batch,y)
dbnormPcom(m)
## End(Not run)
```

---

dbnormSCORE — *Adjusted Coefficient Of Determination for a data normalized for signal drift across batches*

Description

It is a function in dbnorm, a package in R. This function gives a quick notification about the performance of the compiled statistical models namely two-stage regression procedure (see functions such as "ber" and ber-bg using partial bagging model with n=150 bootstrap samples) and/or empirical Bayes methods in two setting of parametric and non-parametric (see "ComBat" in "sva", a package in bioconductor), on accommodation of batch effect. Using this function users will estimate values of adjusted coefficient of determination (Adjusted R- Squared) which address the dependency of each feature (variable) to the batch order in each dataset. Immediately, a score calculated based on the maximum variability estimated by the regression analysis is reported and presented in graph.

This score notifies the consistency of a model performance for the detected features (variables), facilitating quick comparison of the models for selecting one of those models, which is more appropriate to the data structure.

Usage

```
dbnormSCORE(m)
```

Arguments

`m`	A data frame in which rows define the independent experiments (samples) and columns the features (variables), with the batch levels in the first column.

Details

Zero and NA values are not allowed. Optionally missing value can be imputed by `emvf` or `emvd`, functions implemented in `dbnorm` package. Input data must be normalized prior.

Value

Several graphs compiled into a **PDF** file which are a *correlation* plot for each of applied models, a grouped *barplot* presenting the maximum variability associated with batch levels in the raw and the corrected datasets.
Files saved as **csv** in the working directory are a dataset corrected via either of applied models. Also, a two column matrix for Adjusted R-Square for raw and corrected datasets and a table summarizing the score values presented in *barplot*.

References

Giordan (2013) < DOI:10.1007/s12561-013-9081-1 > *https://link.springer.com/article/10.1007/s12561-013-9081-1*
Johnson et al., (2007) < DOI: 10.1093/biostatistics/kxj037 > *http://www.ncbi.nlm.nih.gov/pubmed/16632515*
Leek et al., (2012) < DOI:10.1093/bioinformatics/bts034 > *https://www.ncbi.nlm.nih.gov/pmc/articles/PMC3307112/*

Examples

```
## Not run:
batch<- rep(gl(5,10,labels = c(1:5)),1)
y<- matrix(rnorm(5000),nrow=50)
m<-data.frame(batch,y)
ACDdbnorm(m)
## End(Not run)
```

`emvd` *Estimation of missing value data-based*

Description

This is a function in the `dbnorm`, a package in R. It returns to a matrix of data in which missing values are estimated by the lowest detected value in the entire experiment. By this function, all NA values are replaced by Zero values, that of being ultimately replaced by the lowest value detected in the experiment. Ultimately, data matrix is transposed to restore original structure.

Usage

```
emvd(m)
```

Arguments

`m`	An array or a matrix

Details

empty entries are not allowed

Value

A matrix with estimated missing value.

Examples

```
m<- data.frame(x1=c(50,NA,6,10,30),x2=c(2,8,NA,15,0))
emvd(m)
```

`emvf` *Estimation of missing value feature-based*

Description

This is a function in the `dbnorm`, a package in R. This function returns to a matrix of data in which missing values (Zero and/or NA values) are estimated. By this function, all Zero values are first replaced by NA values, which are then replaced by the lowest detected value on the column margin.

Usage

```
emvf(m)
```

Arguments

`m`	An array or a matrix

Details

empty entries are not allowed

Value

A matrix with estimated missing value.

Examples

```
m<- data.frame(x1=c(50,NA,6,10,30),x2=c(2,8,NA,15,0))
emvf(m)
```

`hclustdbnorm` *Hierarchical clustering analysis of original data and corrected data for batch effect It is a function in* `dbnorm`*, a package in R. This function allows users to evaluate dissimilarity between identical samples (quality control replicates or analytical replicates) analyzed in different batches, prior and after correction using, ber, ber_bg and parametric and non-parametric ComBat . Pearson distance and average method for clustering were considered.*

Description

Hierarchical clustering analysis of original data and corrected data for batch effect It is a function in `dbnorm`, a package in R. This function allows users to evaluate dissimilarity between identical samples (quality control replicates or analytical replicates) analyzed in different batches, prior and after correction using, ber, ber_bg and parametric and non-parametric ComBat . Pearson distance and average method for clustering were considered.

Usage

```
hclustdbnorm(m)
```

Arguments

`m`	A data frame in which rows define the independent experiments (samples) and columns the features (variables), with the batch levels in the first column.

Details

Zero and NA values are not allowed. Optionally missing value can be imputed by the functions such as `emvf` and /or `emvd` implemented in `dbnorm` package. Input must be normalized and transformed prior.

Value

Hierarchical clustering tree for original data (Raw data) and after correction, saved in a single **PDF** file in a working directory and series of **.csv** files includes distance values saved in temporary directory.

References

Johnson et al., (2007) < DOI:10.1093/biostatistics/kxj037 > *http://www.ncbi.nlm.nih.gov/pubmed/16632515*
Leek et al., (2012) < DOI:10.1093/bioinformatics/bts034> *https://www.ncbi.nlm.nih.gov/pmc/articles/PMC3307112/*

Examples

```
## Not run:
batch<- rep(gl(5,10,labels = c(1:5)),1)
y<- matrix(rnorm(5000),nrow=50)
m<-data.frame(batch,y)
hclustdbnorm(m)
## End(Not run)
```

ProfPlotBagging *Profile Plot of Features (variables) in corrected data using ber-bagging model*

Description

It is a function in the dbnorm, a package in R. This function allows users to remove variance associated with batch using “partial bagging” model with n=150 bootstrap samples (see also "ber_bg" function and its package in R). This function visualize the result for global profile of each feature across batches via (*Scatter* plot), (*Violin* plot) and (*Density (or pdf)* plot).
#'

Usage

```
ProfPlotBagging(m)
```

Arguments

m	A data frame in which rows define the independent experiments (samples) and columns the features (variables), with the batch in the first column.

Details

Zero and NA values are not allowed. Optionally missing value can be imputed by the functions such as emvf and/ or emvd implemented in the dbnorm. Input must be normalized and transformed prior.

Value

Original and adjusted datasets in **csv** format together with the series of profile plot for the variables(features) in the sample sets analyzed in the entire experiment provided by the *Scatter* plot,*Violin* plot and *pdf* plot compiled into **PDF** file.

References

M.Giordan (2013) < DOI:10.1007/s12561-013-9081-1 > *https://link.springer.com/article/10.1007/s12561-013-9081-1*

Examples

```
## Not run:
batch<- rep(gl(5,10,labels = c(1:5),1))
y<- matrix(rnorm(5000),nrow=50)
m<-data.frame(batch,y)
profplotber(m)

## End(Not run)
```

`ProfPlotber` *Profile Plot of Features (variables) in ber- corrected data*

Description

It is a function in the `dbnorm`, a package in R. This function allows users to adjust the data for batch effect via location-scale (L/S) model (see also "ber" function and its package in R). This function visualize the result for global profile of each feature across batches via (*Scatter* plot), (*Violin* plot) and (*Density (or pdf)* plot).

Usage

```
ProfPlotber(m)
```

Arguments

`m`	A data frame in which rows define the independent experiments (samples) and columns the features (variables), with the batch in the first column.

Details

Zero and NA values are not allowed. Optionally missing value can be imputed by the functions such as `emvf` and/ or `emvd` implemented in the `dbnorm`. Input must be normalized and transformed prior.

Value

Original and adjusted sets of data in **csv** format together with the series of profile plot for the variables(features) in the sample sets analyzed in the entire experiment provided by the *Scatter* plot,*Violin* plot and *pdf* plot compiled into **PDF** file.

References

M.Giordan (2013) < DOI:10.1007/s12561-013-9081-1 > *https://link.springer.com/article/10.1007/s12561-013-9081-1*

Examples

```
## Not run:
batch<- rep(gl(5,10,labels = c(1:5),1))
y<- matrix(rnorm(5000),nrow=50)
m<-data.frame(batch,y)
profplotber(m)

## End(Not run)
```

`ProfPlotComNPara` *Profile Plot of Features (variables) in corrected data via Non-Parametric ComBat*

Description

It is a function in the `dbnorm`, a package in R. This function allows users to adjust the data for batch effect using non-parametric *Empirical Bayes* approach (see "ComBat" in "sva", a package in bioconductor). *profplotpcom* visualize the result for global profile of each feature across batches via (*Scatter* plot), (*Violin* plot) and (*Density (or pdf)* plot).

Usage

```
ProfPlotComNPara(m)
```

Arguments

`m`	A data frame in which rows define the independent experiments (samples) and columns the features (variables), with the batch in the first column.

Details

Zero and NA values are not allowed. Optionally missing value can be imputed by the functions such as `emvf` and/ or `emvd` implemented in the `dbnorm`. Input must be normalized and transformed prior.

Value

Original and adjusted datasets in **csv** format together with the series of profile plot of the features (variables) in the sample sets provided by *Scatter* plot,*Violin* plot and *pdf* plot compiled into a **PDF** file.

References

Johnson et al., (2007) < DOI:10.1093/biostatistics/kxj037 > *http://www.ncbi.nlm.nih.gov/pubmed/16632515*
Leek et al., (2012) < DOI:10.1093/bioinformatics/bts034> *https://www.ncbi.nlm.nih.gov/pmc/articles/PMC3307112/*

Examples

```
## Not run:
batch<- rep(gl(2,3,labels=(1:2)),2)
y<- matrix(rnorm(6000), nrow=12)
m<- data.frame (batch,y)
profplotnpcom(m)

## End(Not run)
```

`ProfPlotComPara` *Profile Plot of Features (variables) in corrected data via Parametric ComBat*

Description

It is a function in the `dbnorm`, a package in R. This function allows users to adjust the data for batch effect using parametric *Empirical Bayes* approach (see "ComBat" in "sva", a package in bioconductor). *profplotpcom* visualize the result for global profile of each feature across batches via (*Scatter* plot), (*Violin* plot) and (*Density (or pdf)* plot).

Usage

```
ProfPlotComPara(m)
```

Arguments

`m`	A data frame in which rows define the independent experiments (samples) and columns the features (variables), with the batch in the first column.

Details

Zero and NA values are not allowed. Optionally missing value can be imputed by the functions such as `emvf` and/ or `emvd` implemented in the `'dbnorm'`. Input must be normalized and transformed prior.

Value

Original and adjusted sets of data in **csv** format together with the series of profile plot for the features(variables) in the sample sets provided by the *Scatter* plot,*Violin* plot and *pdf* plot compiled into a **PDF** file.

References

Johnson et al., (2007) < DOI:10.1093/biostatistics/kxj037 > *http://www.ncbi.nlm.nih.gov/pubmed/16632515*
Leek et al., (2012) < DOI:10.1093/bioinformatics/bts034> *https://www.ncbi.nlm.nih.gov/pmc/articles/PMC3307112/*

Examples

```
## Not run:
batch<- rep(gl(2,3,labels=(1:2)),2)
y<- matrix(rnorm(6000), nrow=12)
m<- data.frame (batch,y)
profplotpcom(m)

## End(Not run)
```

`ProfPlotraw` *Profile Plot of Features (variables) in original data (Raw data)*

Description

It is a function in the dbnorm This function informs you about the presence of across batch signal drift or batch effect in the raw data determined by the shifted probability density function plots (*pdf* plots) of features (variables) detected in an experiment.

Usage

```
ProfPlotraw(m)
```

Arguments

m	A data frame in which rows define the independent experiments (samples) and columns the features (variables), with the batch level in the first column.

Details

Zero and NA values are not allowed. Optionally missing value can be imputed by functions such as emvf or emvd Compiled in the 'dbnorm' package. Input must be normalized and transformed prior.

Value

Original dataset in **csv** format together with the series of profile plot for the features (variables) in the sample sets analyzed in the entire experiment provided by the *Scatter* plot,*Violin* plot and *pdf* plot compiled into **PDF** file.

Examples

```
## Not run:
batch<- rep(gl(5,10,labels = c(1:5),1))
y<- matrix(rnorm(5000),nrow=50)
m<-data.frame(batch,y)
profplotraw(m)

## End(Not run)
```

`Visodbnorm` *Visualization and normalization of signal drift across batches*

Description

This function performs batch effect adjustment via three statistical models, namely two-stage procedure as described by M. Giordan (2013) < DOI:10.1007/s12561-013-9081-1> (see also "ber" and ber_bg) and/or empirical Bayes methods in two setting of parametric and non-parametric as described by Johnson et al., (2007) < DOI: 10.1093/biostatistics/kxj037 > (see "comBat" in "sva", a package in bioconductor) . Meanwhile, the graphical inferences in the context of unsupervised learning algorithms create visual inspection to inform users about the spatial separation of the sample sets analyzed in the different analytical runs alongside the distribution of the features (variables) in the sample sets and across multiple batches. For bagging model, partial bagging model with n=150 bootstrap samples is considered.

Usage

```
Visodbnorm(f)
```

Arguments

`f`	A data frame in which rows define the independent experiments (samples) and columns the features (variables), with the batch in the first column.

Details

Zero and NA values are not allowed. Optionally missing value can be imputed by `emvf` and /or `emvd`, functions implemented in the `dbnorm` package. Input data must be normalized prior.

Value

Three datasets, adjusted by either of applied statistical algorithms prepared in **csv** and together with series of plot such as *PCA* plot and *Scree plot* compiled into a **PDF** file are saved in the working directory. *RLA* plots are represented in the **Viewer** panel of **rstudio**.

References

M.Giordan (2013) < DOI:10.1007/s12561-013-9081-1 > *https://link.springer.com/article/10.1007/s12561-013-9081-1*

Johnson et al., (2007) < DOI:10.1093/biostatistics/kxj037> *http://www.ncbi.nlm.nih.gov/pubmed/16632515*

Examples

```
## Not run:
Visdbnorm
batch<- rep(gl(5,10,labels = c(1:5)),1)
y<- matrix(rnorm(5000),nrow=50)
f<-data.frame(batch,y)
Visdbnorm(f)
## End(Not run)
```

Index

∗ **Adjusted**
dbnormSCORE, 6
∗ **ComBat-Parametric**
dbnormPcom, 5
∗ **ComBat**
dbnormNPcom, 4
ProfPlotComNPara, 12
ProfPlotComPara, 13
∗ **Missing**
emvd, 7
emvf, 8
∗ **NON-Parametric**
dbnormNPcom, 4
∗ **Non**
ProfPlotComNPara, 12
∗ **Parametric**
ProfPlotComPara, 13
∗ **R-squared**
dbnormSCORE, 6
∗ **Unsupervised**
dbnormBagging, 2
dbnormBer, 3
∗ **Visualization**
Visodbnorm, 15
∗ **across**
Visodbnorm, 15
∗ **analysis**
dbnormBagging, 2
dbnormBer, 3
dbnormNPcom, 4
dbnormPcom, 5
∗ **and**
dbnormBagging, 2
dbnormBer, 3
dbnormNPcom, 4
dbnormPcom, 5
ProfPlotBagging, 10
ProfPlotber, 11
ProfPlotComPara, 13
Visodbnorm, 15
∗ **batch**
Visodbnorm, 15
∗ **ber-model**
dbnormBagging, 2
dbnormBer, 3
∗ **ber**
ProfPlotBagging, 10
ProfPlotber, 11
∗ **correction**
ProfPlotBagging, 10
ProfPlotber, 11
∗ **data**
dbnormBagging, 2
dbnormBer, 3
dbnormNPcom, 4
dbnormPcom, 5
ProfPlotraw, 14
∗ **estimation**
emvd, 7
emvf, 8
∗ **for**
dbnormBagging, 2
dbnormBer, 3
dbnormNPcom, 4
dbnormPcom, 5
∗ **hclust**
hclustdbnorm, 9
∗ **normalization**
Visodbnorm, 15
∗ **normalized**
dbnormBagging, 2
dbnormBer, 3
dbnormNPcom, 4
dbnormPcom, 5
∗ **parametric**
ProfPlotComNPara, 12
∗ **plot**
ProfPlotBagging, 10
ProfPlotber, 11
ProfPlotComNPara, 12
ProfPlotComPara, 13
ProfPlotraw, 14
∗ **profile**
ProfPlotBagging, 10
ProfPlotber, 11
ProfPlotComNPara, 12

ProfPlotComPara, 13
ProfPlotraw, 14
∗ **raw**
ProfPlotraw, 14
∗ **regression**
dbnormBagging, 2
dbnormBer, 3
dbnormNPcom, 4
dbnormPcom, 5
∗ **unsupervised**
dbnormNPcom, 4
dbnormPcom, 5
∗ **value**
emvd, 7
emvf, 8
∗ **via**
dbnormBagging, 2
dbnormBer, 3
dbnormNPcom, 4
dbnormPcom, 5

dbnormBagging, 2
dbnormBer, 3
dbnormNPcom, 4
dbnormPcom, 5
dbnormSCORE, 6

emvd, 7
emvf, 8

hclustdbnorm, 9

ProfPlotBagging, 10
ProfPlotber, 11
ProfPlotComNPara, 12
ProfPlotComPara, 13
ProfPlotraw, 14

Visodbnorm, 15
